# 2-Step Drop Impact Analysis of a Miniature Mobile Haptic Actuator Considering High Strain Rate and Damping Effects

**DOI:** 10.3390/mi10040272

**Published:** 2019-04-23

**Authors:** Byungjoo Choi, Hyunjun Choi, You-sung Kang, Yongho Jeon, Moon Gu Lee

**Affiliations:** Department of Mechanical Engineering, Ajou University, Su-won Si 16499, Korea; dasom@ajou.ac.kr (B.C.); reset0331@gmail.com (H.C.); gidalim89@ajou.ac.kr (Y.-s.K.); princaps@ajou.ac.kr (Y.J.)

**Keywords:** haptic actuator, impact analysis, high strain rate effect, damping, 2-step analysis

## Abstract

In recent times, the haptic actuators have been providing users with tactile feedback via vibration for a realistic experience. The vibration spring must be designed thin and small to use a haptic actuator in a smart device. Therefore, considerable interests have been exhibited with respect to the impact characteristics of these springs. However, these springs have been difficult to analyze due to their small size. In this study, drop impact experiments and analyses were performed to examine the damages of the mechanical spring in a miniature haptic actuator. Finally, an analytical model with high strain rate and damping effects was constructed to analyze the impact characteristics.

## 1. Introduction

A haptic actuator is a component in mobile devices used to transmit tactile signals to the users. The main function of this component is to vibrate, when the mobile device receives messages, e-mails, social network service notifications, and so on. High definition haptic technology has been developed in recent years to transmit a host of dynamic expressions to the user. To realize this, a thin spring is required together with a strong electromagnet for achieving a high acceleration and wide frequency band. However, springs that deliver a realistic tactile feeling are less durable, as they are thin and structurally weak. In particular, drop impact-induced deformation is a typical type of damage observed in the springs, and several studies are being conducted in the industry to analyze this aspect. Due to the high velocity and small size, it is difficult to observe the impact behavior and identify the vulnerabilities of the springs. Therefore, a new analytical approach is required for the design that fulfils the reliability criterion of acceleration variation, which is only 10% when falling at 1.8 m. 

Prior academic research has mainly focused on the drop impact tests and analysis of the finished product. The product level differs from the parts level, as it considers the key factors, such as the strain rate and damping modeling, in detail. However, the findings of some of these studies are be summarized to understand the research direction and analysis method.

Goyal et al. [1] designed a new impact tester and analyzed the multiple impacts of portable electronic products. They constructed an automated system for repeatability, which demonstrated that the impact direction could be regulated while fulfilling the free-drop conditions.

Lim et al. [2] used ABAQUS/Explicit to investigate the drop impact of an electronic pager. They analyzed the strain and impact force of the pager housing and verified the exceptional correlation between the results of the physical experiment and the computer-simulated analysis. Furthermore, they determined the impact direction and height that could result in critical damages.

Zhu [3] performed the finite element analysis to evaluate the reliability of a mobile phone. In this work, three impact modes (disengagement of the battery snap fit, preload spring failure, and ball grid array (BGA) solder crack) were numerically modeled and their correlation with the experiment was studied. Finally, the strain rate effect, impact contact force, and impact acceleration were individually confirmed in each mode.

Kim et al. [4] performed the drop impact simulation of a cell phone using the LS-DYNA explicit code. To verify the analytical model experimentally, both the global and local impact responses were confirmed with a high-speed camera. They also predicted the potential damage of the mobile phone using a statistical analysis in their experiment.

Karppinen et al. [5] compared the product level and board level drop tests, because the drop tests of handheld products demonstrated different results depending on the enclosure, impact orientation, strike surfaces, and mounting of the board. They also compared the mechanical impact responses, which were measured by laser vibrometry, and the acoustic excitation method was used for the analysis. Though the impact load delivered to the board was different in each test, the default failure mode was observed to be the same.

Mattila et al. [6] evaluated the drop impact responses of eight smartphones from various manufacturers. A small accelerometer and a strain gauge were attached inside the smartphones to measure their drop impact response. The maximum strain, average of the maximum strain rate, frequency of the mode shape, and the maximum deceleration were calculated for each product. 

**Figure 1:**
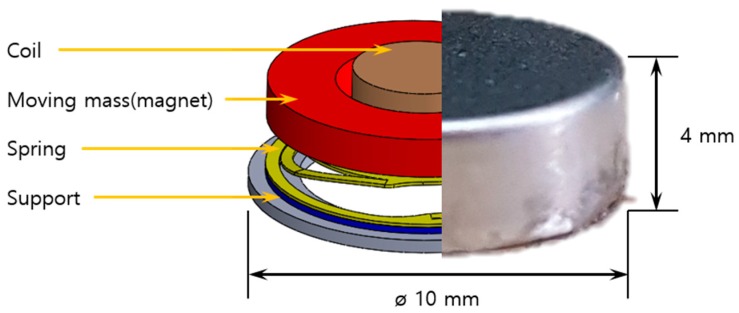
A haptic actuator for a smart device.

The haptic actuator spring is 9 mm in diameter and 0.3 mm in thickness and weighs only 0.1 g (Figure 1). The moving mass, which is approximately twice that of the spring mass, is attached to the spring resulting in serious damage after the primary impact due to an internal secondary impact. Choi et al. conducted a study related to the drop impact of haptic actuators [7]. They developed their finite element model using material properties, such as the microtensile strength and damping ratio, and compared the impact deformation and force using a drop test. However, research on the high strain rate effect and damping modeling, which can be used to improve the accuracy of an analytical model, are limited.

In this study, the Johnson–Cook model considering wave propagation was applied considering a high strain rate. A 2-step analysis was conducted for improving the calculation efficiency, and damping was modeled for each stage. Consequently, an analytical model was proposed after comparing the results from the analysis of a miniature haptic actuator with the respective experimental values.

## 2. Material Modeling

### 2.1. Haptic Spring

Regarding drop impact, a typical smartphone collides with the ground at a velocity of 3–6 m/s, depending on the initial height in the range of 0.5–1.8 m, and the inner elastic parts deform at higher velocities. In other words, parts that induce elastic deformation, such as a spring, must be modeled considering the strain rate effect. The ANSYS LS-DYNA utilizes the Johnson–Cook model [8] for modeling the strain rate effect, and the constitutive equation is presented as follows.

(1) $$\sigma =\left(A+B\varepsilon^{n}\right)\left(1+C\ln\dot{\varepsilon }\right)$$

The first term represents the strain-hardening effect of the material, and the strain–stress curve is expressed by the Ludwick equation [9]. A is the initial yield strength, B is the strength coefficient, ε is the plastic strain, and n is the strain-hardening exponent. The second term represents the strain hardening caused by the strain rate effect. $\dot{\varepsilon }$ is the strain rate, and C represents the strength at which the strain rate varies.

The first term refers to the coefficient obtained by fitting a stress–strain curve plotted according to a quasi-static tensile test. The constant C from the second term can be calculated based on the stress–strain curve measured at various strain rates in the split Hopkinson pressure bar (SHPB) test proposed by Kolsky [10]. A round bar-type specimen is required in the test, as the SHPB test measures the deformation caused by an elastic wave between the input and output rods.

**Figure 2:**
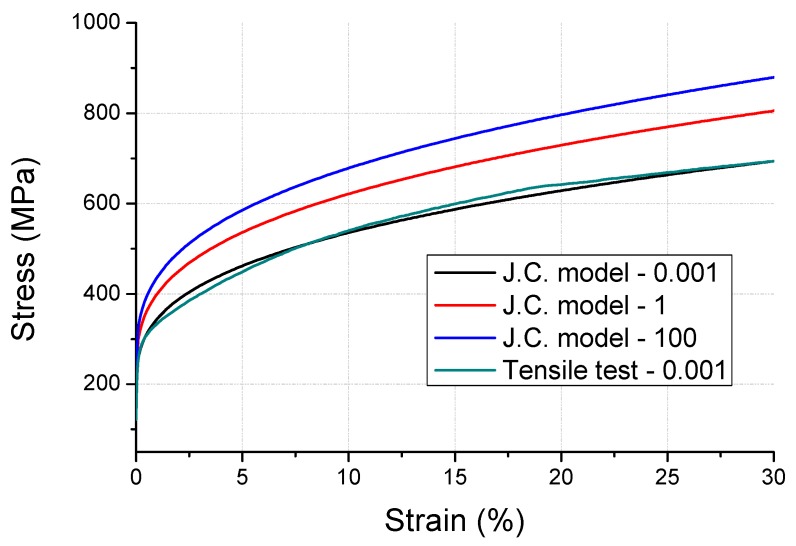
Stress–strain curve of SUS304 based on strain rate variations.

**Figure 3:**
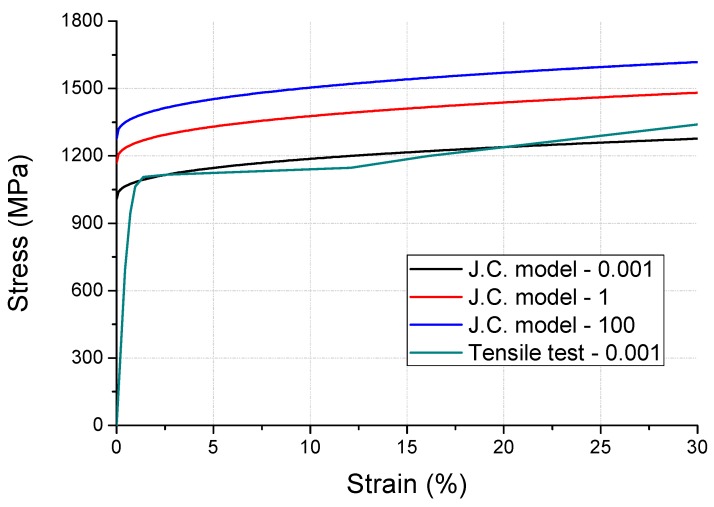
Stress–strain curve of SUS301 based on strain rate variations.

However, to enhance the durability of SUS301, the composition of chrome and nickel in SUS304 was reduced and cold-rolled as a plate. Therefore, we could not obtain round bar specimens for the SHPB tests, because only a thin sheet material resulted. To overcome this limitation, firstly, the Johnson–Cook model was constructed on the round bar specimens of SUS304 manufactured with similar materials and processes (Figure 2). The value of the constant C is 0.02 for SUS304, which was applied to SUS301, and the Johnson–Cook model was developed. The stress–strain curve of SUS301 is shown in Figure 3. We used the induced SUS301 model from the experimental SUS304 model. The stress–strain curve was derived from the research of Lee et al. [11]. A mismatch observed in the graph between the curves of Johnson Cook model ($\dot{\varepsilon }=0.001)$ and tensile test ($\dot{\varepsilon }=0.001)$ in the increase of the initial stress was compensated by using the modulus values in the analysis. The constants of the reference material SUS304 and spring material SUS301 used in the Johnson–Cook model are summarized in Table 1.

### 2.2. Other Components

The other components determine the impact force delivered to the spring of the haptic actuator, and therefore, a detailed material modeling was required. The dummy mass comprised aluminum and polycarbonate, and the vibration motor comprised of a housing with a moving mass. The Johnson–Cook model was also applied to the parts absorbing the energy transmitted to the spring due to plastic deformation. The used material models of the dummy (Al6061-T6) and the sensor head (stainless steel) were from the ANSYS library and their constants are summarized in Table 2.

The impact analysis produced dominant results on the velocity and mass of the drop object. The initial velocity was 5.88 m/s when impacting the ground from a height of 1.8 m. This was simply applied to the boundary condition. However, the mass required relatively detailed modeling. Firstly, the mass of polycarbonates attached to aluminum affected the occurrence of the primary impact in the dummy. Secondly, the moving mass attached to the spring affected the internal impact to the housing. The mass of the specimen, including the haptic actuator, was 114 g. The material properties of each of the specimens are summarized in Table 3.

The contact between the parts was modeled under two conditions, including “slightly expected slip” and “fixed with no slip”. 

The former used the condition of “frictional contact”. A static coefficient of 0.61 and a dynamic coefficient of 0.47 between aluminum and mild steel were applied at the interface between the dummy and sensor head for the external impact, while a static coefficient of 0.74 and a dynamic coefficient of 0.57 among the mild steel were applied at the interface between the haptic housing and moving mass for the internal impact. However, the analysis results were not affected to the friction coefficient, as there was no contact sliding during the impact behavior. 

The latter used the condition of “bonded”. The haptic actuator was fixed by spot welding the parts together. However, commonly used bonded conditions were applied to the interfaces, as the exact welding contact stiffness were not be known.

## 3. 2-Step Analysis Modeling

The impact analysis using the explicit solver required considerable amount of computation time and resources as it involved several iterations. When an electronic device is analyzed, the impact is first applied to the outside and then transmitted to the inside. Therefore, a full-scale analysis requires considerable amount of time to calculate these two impacts in the transmission process. The calculation of a single drop impact of the haptic actuator specimen required 6 h and used five CPU cores. However, dividing the impact into separate external and internal impacts and analyzing them as two steps reduced the calculation time to 0.5 h each. This method can be used for efficient and repeated analysis by changing the 3D model or material properties in the design process.

**Figure 4:**
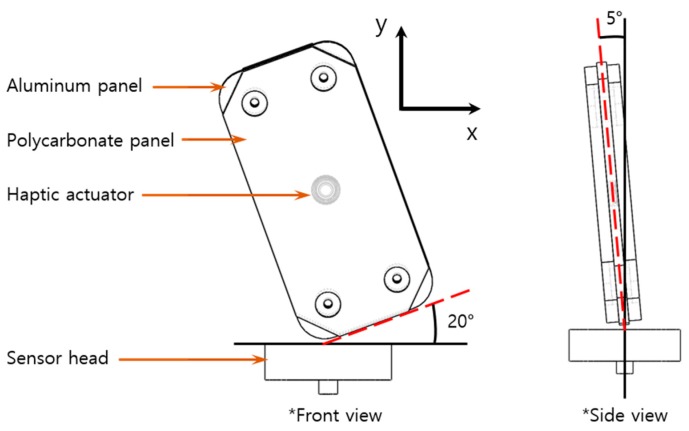
Drop impact analysis model.

The drop impact model of the haptic actuator is shown in Figure 4. The specimen was modeled using a 20° tilt from the sensor surface to simulate an experiential worst case of the impact force. Under this condition, the center of gravity of the dummy phone was perpendicular to the ground. In addition, there was almost no drop impact under perpendicular to the ground. Therefore, the model was modified by titling the specimen sideways by 5°. This combination was applied, as these two angles (20° and 5°) were most frequently observed in a series of repeated impact tests.

### 3.1. First Step—External Impact

The purpose of this stage was to extract the velocity history of the attached surface of the haptic actuator, when the specimen collided with an impact force sensor (PCB, ICP® quartz force sensor, 200C50).

**Figure 5:**
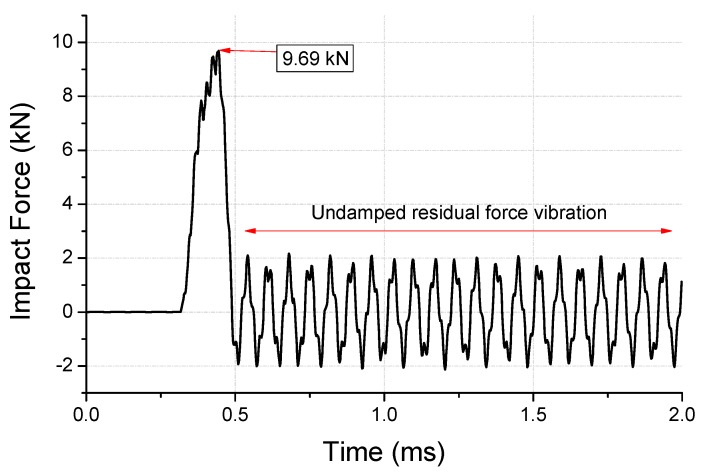
Analysis of the undamped impact force of first-step drop.

**Figure 6:**
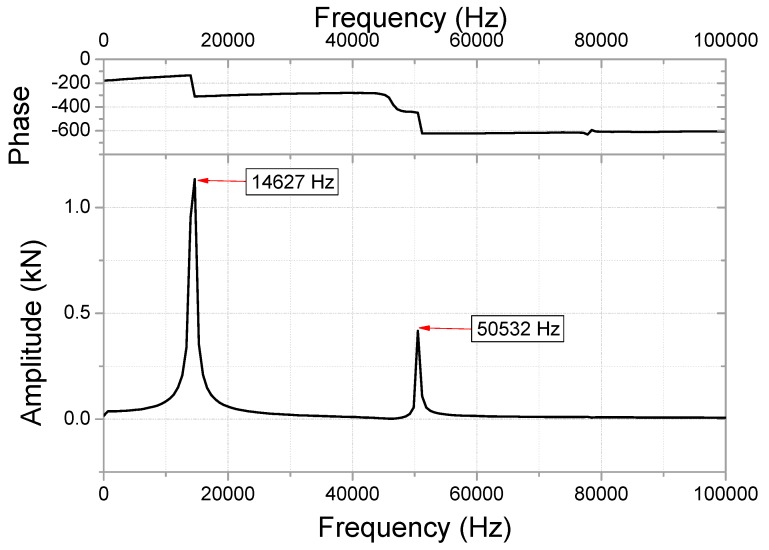
Fast Fourier transform (FFT) for residual force vibration (undamped area shown in Figure 5) in the frequency domain.

The impact force for the initial analytical model is shown in Figure 5. A peak force of 9.69 kN is observed in this figure; however, an undamped residual force vibration was confirmed. The abnormal attenuation was controlled by damping modeling, as energy dissipation could result in an abnormal rise in stress. The residual force vibration can be used to discover the dominant frequencies with high amplitude using fast Fourier transform (FFT), as shown in Figure 6. The modeling was performed to attenuate the main vibration frequency (14627 Hz) of the residual force vibration.

**Figure 7:**
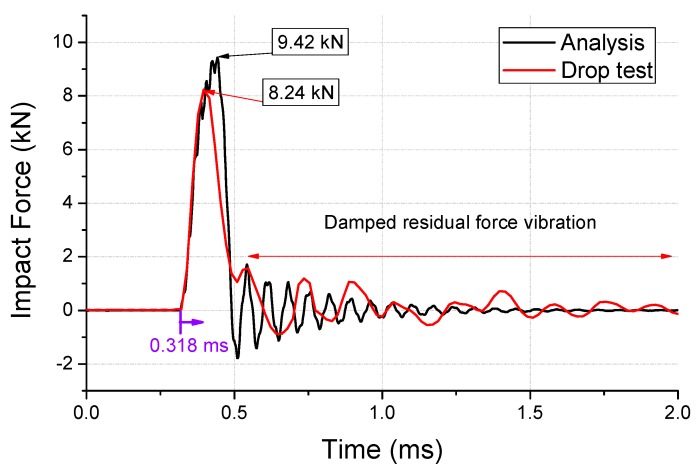
Damped impact force of the first-step drop-test and analysis.

The impact force measured in the drop test is shown in Figure 7. The impact force is estimated to be 8.24 kN, when the specimen collides with the impact force sensor. The second and third peak forces in the residual force vibration are observed to be 1.58 and 1.19 kN, respectively. These values are used in the computation of the damping coefficient (ξ = 0.045) using the logarithmic decrement method.

**Figure 8:**
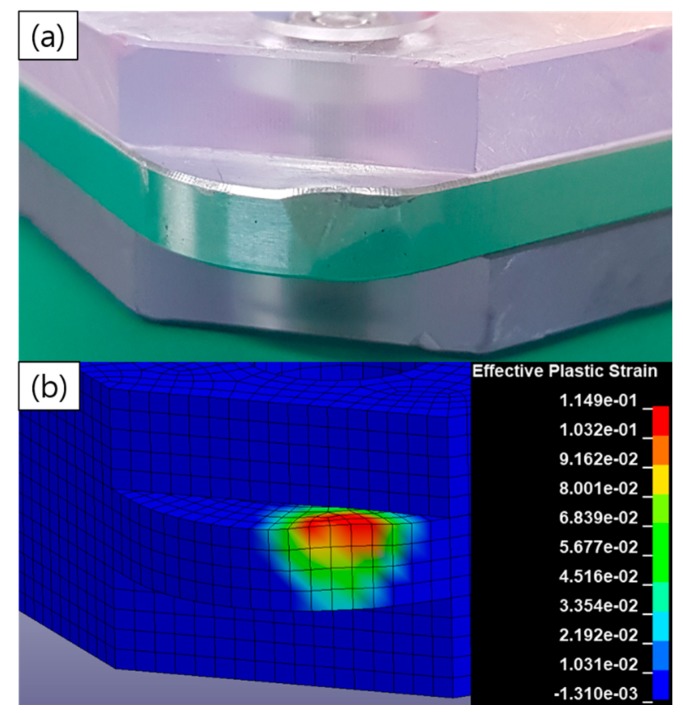
Plastic strain of the dummy after (**a**) the impact test and (**b**) analysis.

The Rayleigh damping C used in the analysis is defined as follows [12]:

(2) C=αM+βK,

where M is the mass matrix, K is the stiffness matrix, and α and β are constants. As the dominant frequency of the residual force vibration and the damping ratio were measured during the test, the value of the constant α can be calculated as follows:

(3) α=2ξω,

where the value of α is 8301. The analyzed impact force obtained by applying this value is shown in Figure 7. The peak value of the force is observed to be 9.42 kN, which is 3% less than that before the damping modeling. An error of 14% resulted from the test, because the Johnson–Cook model of the aluminum dummy was not measured. Figure 8 compares the impact strain from the test and plastic strain from the analysis. It is evident from this figure that the type of deformation observed during the test agrees well with that observed during the analysis, and the maximum strain of 0.11 was confirmed by the analysis.

### 3.2. Second Step—Internal Impact

The purpose of this stage was to identify the stress distribution and plastic strain in the haptic actuator based on the velocity history obtained from the first step.

**Figure 9:**
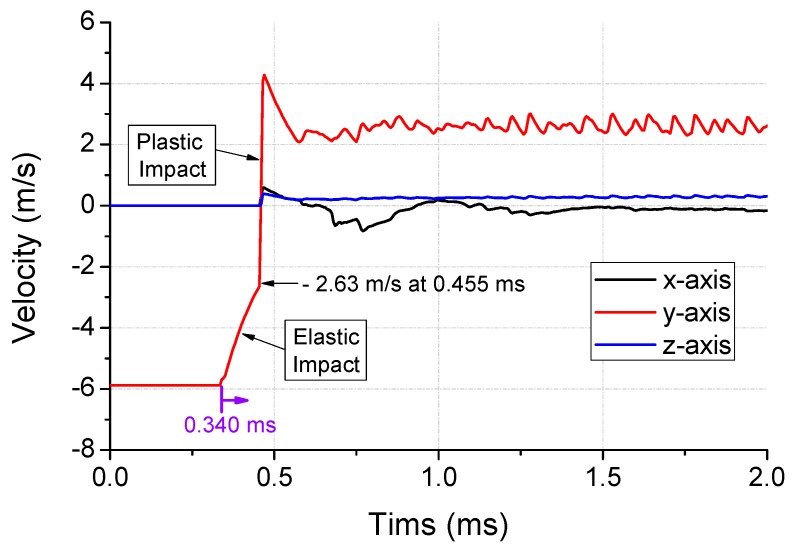
Velocity profile of haptic attachment surface to dummy in first-step analysis.

The x-, y-, and z-axis velocity history of the haptic actuator attached to the surface of the dummy is shown in Figure 9. All the components initiate the drop at an initial velocity of −5.88 m/s in the y-axis. Therefore, no disturbance was experienced before the impact. The event is initiated at 0.340 ms, and the occurrence of elastic impact is observed over 0.115 ms. After a threshold of −2.63 m/s, the plastic impact is observed at the maximum repulsion rate of +4.27 m/s for a short duration of 0.01 ms. The y-axis velocity converges to an average of 2.57 m/s after the loss of contact, while the starting point of the velocity variation demonstrates a time delay of 0.022 ms from the impact contact of the dummy.

**Figure 10:**
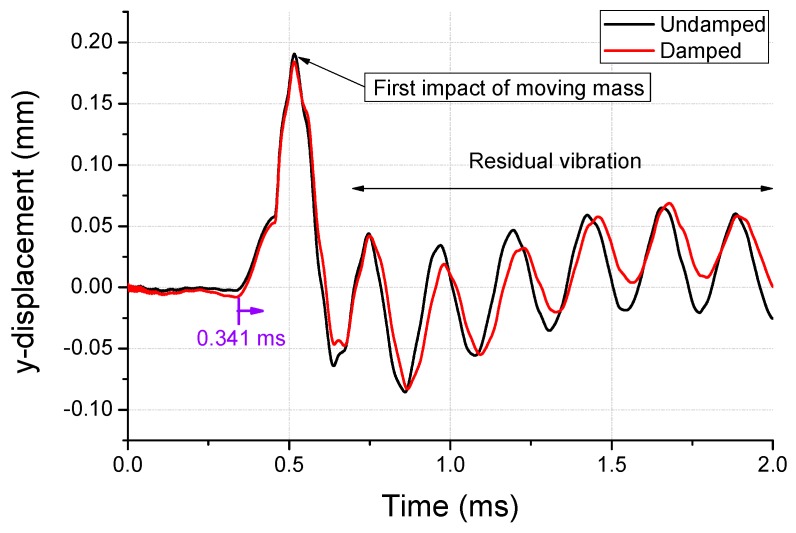
Y-displacement of the spring with and without damping in the second-step analysis.

**Figure 11:**
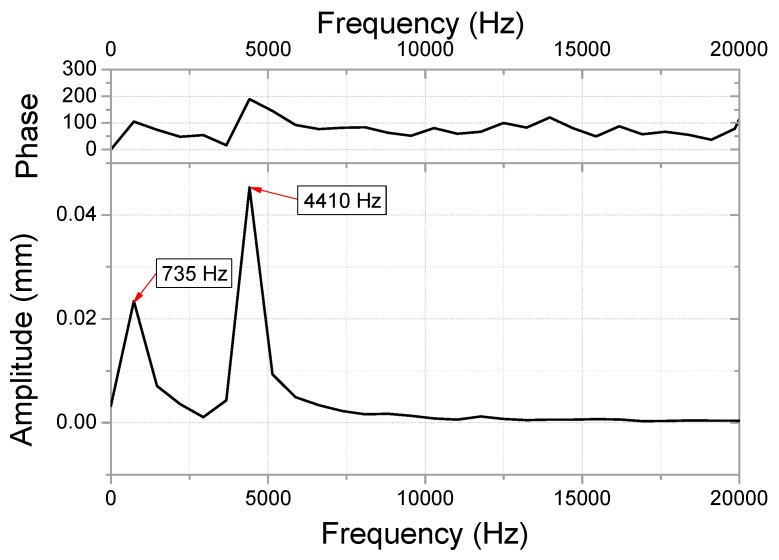
FFT for residual vibration (residual vibration area from Figure 9) in the frequency domain.

**Figure 12:**
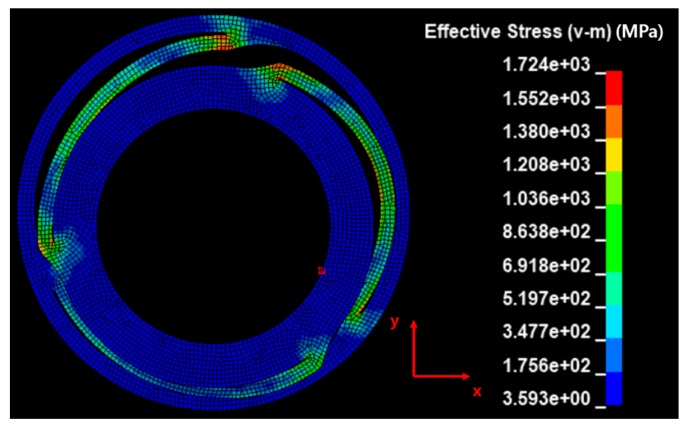
Effective stress of haptic spring at the moment of impact.

Generally, springs demonstrate a minimal amount of damping, as they induce elastic deformation for mechanical purposes. However, during the analysis of the input velocity history, the undamped spring (shown in Figure 10) exhibited no attenuation in residual vibration. Therefore, an FFT was performed in the frequency domain for modeling the damping. The results from this FFT are shown in Figure 11. At low frequencies, a giant wave is generated with high frequency vibrations. The damping was modeled using a high frequency (4410 Hz), which was dominant during the energy dissipation. The damping ratio (ξ) required for the modeling was 0.02, which was based on authors’ previous measurements [7]. The constant α for the mass matrix is calculated as 1180 using Equation (3). The y-displacement of the damped spring is shown in Figure 10. The maximum equivalent stress of the spring is estimated to be 1803 MPa while undamped, which declines by 4% to 1724 MPa after the damping modeling. The moving mass attached to the spring collided with the housing in the −y direction, and this moment of stress distribution is shown in Figure 12. A high stress distribution of approximately 1700 MPa is observed in the upper notch, where tensile stress is applied to the spring. In contrast, a relatively low stress distribution of approximately 1200 MPa is observed in the lower notch. The figure shows four sites, in which the damage is expected to exceed the yield stress of 1377 MPa. The yield stress is observed to be 18% higher than 1170 MPa at a strain rate of 0.001/s (shown in Figure 3) in the tensile tests.

**Figure 13:**
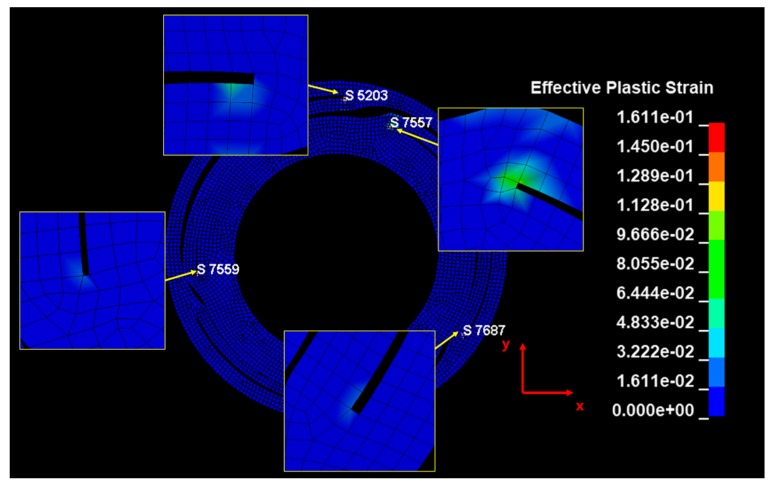
Effective plastic strain of haptic spring at steady state after impact (with number of elements).

**Figure 14:**
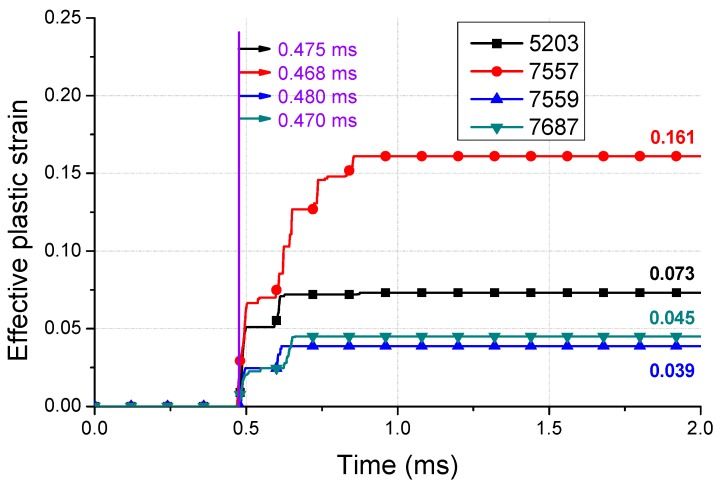
Effective plastic strain of the damage area (with the number of elements shown in Figure 10).

The effective plastic strain distribution of the spring is shown in Figure 13. At each position exceeding the yield, a maximum of 0.161 strain is observed for 3–6 elements, each with a size of 0.1 mm. The quantitative plastic strain of each element is shown in Figure 14. The occurrence of the plastic strain is observed mainly in the spiral notch region, where the upper and lower plates are connected. The upper site (+y direction in Figure 13) demonstrates a higher strain and wider stress distribution. In addition, it was confirmed that the notch connected to the upper plate demonstrated a higher plastic strain than the one in the lower plate, because the moving mass caused a secondary impact on the inner housing. The concentration of the plastic strain in the local region was confirmed by this analysis. If the impact load was applied repeatedly, the plastic deformation would increase possibly resulting in malfunction.

## 4. Drop Impact Tests

The haptic actuator must express a wide frequency at an acceleration of 0.5 g or more to deliver various tactile sensations. If the frequency history of the vibration signal changes, the operation of delivering a haptic expression to the user becomes impossible. More precisely, a vibration frequency and acceleration change of higher than 10% due to drop impact is regarded as a malfunction. Experiments were performed to determine the possibilities of malfunction resulting from a series of drop impacts, and the specimen was dropped from a height of 1.8 m to compare the results with those from the analysis. Two specimens were used in the drop tests, and the signals were measured with an acceleration sensor (PCB, Ceramic shear ICP® accel, 333B50) before and after the drop impact.

### 4.1. Driving Acceleration of the Haptic Actuator

**Figure 15:**
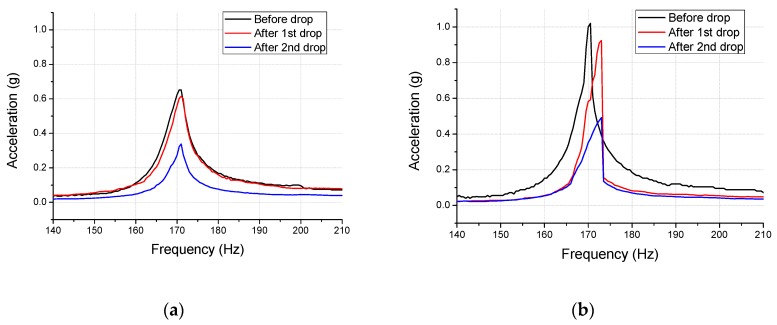
Comparison of the vibration acceleration of the haptic actuator before and after the drop impact tests (**a**) No. 1 specimen (**b**) No. 2 specimen.

A comparison of the vibration acceleration values of the haptic actuator before and after the drop impact tests is shown in Figure 15, and the quantitative data are summarized in Table 4. It is observed from the figure that the peak acceleration and frequency band of the No. 1 specimen decline by 5% and 29% after the first impact. However, no change is observed in the impact damage, as it is within the range of the measurement error. However, after the secondary drop impact, the acceleration decreases by 48%, and the frequency band decreases by 0.5 g owing to the damages in the spring. The acceleration of the No. 2 specimen reduces by 10% after the first impact and the frequency increases from 170.5–173 Hz. Moreover, the band width decreases by 22% after the first drop. The acceleration declined by 52% owing to the considerable amount of damage in the spring after the second drop, and the frequency band decreases to less than 0.5 g. The peak acceleration of the No. 2 specimen demonstrates a sharp decrease after the first impact due to the interference of the moving mass with adjacent components, which results from the spring deformation.

### 4.2. Correlation between the Analysis and Tests

The concentrated damage on the spring notch was confirmed by the single drop impact analysis (shown in Figure 13). The experimental results demonstrated that the vibration frequency was almost unchanged in the No. 1 specimen, while a variation was observed in case of No. 2 specimen. This difference can be explained using two factors. Firstly, the number of variables limited in the analysis but the impact tests were increased. In other words, it is necessary to increase the number of specimens to eliminate the uncertainty. It is also important to supplement the study with statistical analysis. Secondly, the plastic deformation in the analysis was limited to a minor area of the notch, which implies the possibility of no variations in the vibration characteristics of the spring. However, if the stress and strain of the spring were repeated during the impact analysis irrespective of this fact, the vibration characteristics could change due to the accumulation of damages.

## 5. Conclusions

The drop impact experiments and analyses were performed to examine the spring damage of a miniature haptic actuator. The results obtained were as follows:

(1) The Johnson–Cook model was applied to the drop impact analysis of the haptic spring considering a high strain rate effect, and the computation time of the 2-step analysis was observed to be 86% less than that of the single-step analysis.
(2) The attenuation of the signal measured by the impact force sensor was applied to precisely model the impact force in the first-step analysis. The plastic strain of the impact contact area obtained from the analysis and experiment agreed well; however, the peak force was observed to be 14% higher in the analysis than in the experiment. The Johnson–Cook model of an aluminum dummy was required to improve the accuracy of the analysis.
(3) The damping coefficient (ξ) measured in the authors’ previous study was used to model the damping of the spring in the second-step analysis. The occurrence of the maximum stress of the SUS301 spring (1724 MPa) and the greater-than-yield stresses of 1377 MPa at the four notches of the spring was confirmed using the strain constant C of SUS304. The maximum generated plastic strain of 0.161 was the same as the highest stress area.
(4) The acceleration changes after the first drop impact were different across the two specimens in the tests. The difference could be because of the increase in the number of variables in these experiments than analysis. Moreover, the plastic strain in the microscopic region may not possibly generate a variation in experimental acceleration. Due to the limited number of specimens used for the drop test, the statistical analysis of a greater number of experiments is needed for improved accuracy.

In conclusion, an analytical model with a high strain rate and damping effects was successfully constructed for analyzing the impact characteristics of the miniature haptic actuator.

## Figures and Tables

**Table 1:** Constants of Johnson–Cook model for the haptic spring.

Parameters	SUS304	SUS301
A (Initial yield strength)	215 MPa	1170 MPa
B (Hardening constant)	1945 MPa	1140MPa
n (Hardening exponent)	0.34	0.37
C (Strain constant)	0.02	0.02

**Table 2:** Constants of Johnson–Cook model for the dummy and sensor.

Parameters	Al6061-T6	Stainless Steel
A (Initial yield strength)	270 MPa	792 MPa
B (Hardening constant)	138 MPa	510 MPa
n (Hardening exponent)	0.18	0.26
C (Strain constant)	0.002	0.014

**Table 3:** Elastic and physical properties of the specimens used in the drop impact model.

Part	Material	Density (kg/m^3^)	Bulk Modulus (GPa)	Shear Modulus (GPa)	Mass (g)
Spring	SUS301	7850	152	78	0.10
Moving mass	Stainless steel 4340	7830	159	81	0.19
Haptic housing	Stainless steel 4340	7830	159	81	0.41
Dummy body	Aluminum 6061-T6	2804	69	26	53.86
Dummy cover	Polycarbonate	1300	2	1	29.57
Sensor head	Stainless steel	7850	169	73	155.29

**Table 4:** Acceleration and frequency band variations due to the drop impact tests.

Specimen	Peak Acceleration (g)			Band Width at 0.5 g (Hz)		
	Before Drop	After 1st Drop	After 2nd Drop	Before Drop	After 1st Drop	After 2nd Drop
No. 1	0.65 at 171 Hz	0.62 at 171 Hz	0.34 at 171 Hz	3.5	2.5	0
No. 2	1.02 at 170.5 Hz	0.92 at 173 Hz	049 at 173 Hz	4.5	3.5	0
